# Radical resection of T1 pancreatic adenocarcinoma with a pseudocyst of the tail due to acute obstructive pancreatitis: report of a case

**DOI:** 10.1186/s40792-016-0268-9

**Published:** 2016-11-30

**Authors:** Yuki Fujiwara, Fumitake Suzuki, Masaru Kanehira, Yasuro Futagawa, Tomoyoshi Okamoto, Katsuhiko Yanaga

**Affiliations:** 1 Department of Surgery, The Jikei Daisan Hospital, 4-11-1, Izumihoncho, Komae-shi, Tokyo 201-8601 Japan; 2 Department of Surgery, The Jikei University School of Medicine, Tokyo, Japan

**Keywords:** Small pancreatic cancer, Pancreatic pseudocyst, Acute pancreatitis

## Abstract

A 53-year-old male visited his primary physician for epigastric and back pain. Abdominal-enhanced computed tomography (CT) revealed a simple cyst of the pancreatic tail attached to the stomach. A distal main pancreatic duct (MPD) was clearly dilated, but no pancreatic tumor was identified around the stenosis of MPD by CT scan and magnetic resonance cholangiopancreatography (MRCP). Endoscopic retrograde pancreatography (ERP) revealed stenosis and distal dilation of the MPD located between the body and tail of the pancreas. Endoscopic ultrasound (EUS) revealed a low density mass of 7 mm in size with distal dilation of the MPD. With the suspicion of a small pancreatic cancer, the patient underwent distal pancreatectomy and splenectomy with lymph node dissection (D2). On histopathological evaluation, a small pancreatic adenocarcinoma of 6 mm in size was detected around the stenosis of MPD. Final pathological diagnosis was moderately differentiated invasive ductal adenocarcinoma of the pancreas with no lymph node metastasis (Japan Pancreatic Society (JPS) classification 7th edition; Pbt, TS1 (6 mm), tub2, intermediate type, INF β, ly1, v1, ne1, mpd(-), pT1b, pN0, pM0, stage IA,PCM(-), DCM(-) and the Union International Control Cancer (UICC) classification of malignant tumors 6th edition; pT1, pN0, pM0, stage IA, R0). We herein reported a patient who underwent radical resection for T1 pancreatic adenocarcinoma of 6 mm in diameter which caused acute pancreatitis and a pseudocyst due to obstruction of the MPD.

## Background

Pancreatic cancer is one of the most frequent malignant tumors in the world. An overall survival rate remains poor because of advanced stage at the time of diagnosis, and rapid tumor growth despite the improvement in imaging studies [1]. Surgical cases of tumor factor 1 (T1) in patients with pancreatic cancer are rare which are 2.95% in the literature, of which the tumors less than 10 mm in size account for only 0.55% in patients with resectable pancreatic cancer [2]. We herein reported a radical resection of patients with a 6 mm T1 pancreatic adenocarcinoma which prevented clinically as a pseudocyst due to obstructive acute pancreatitis.

### Case presentation

**Fig. 1:**
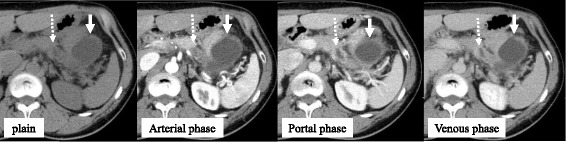
Abdominal-enhanced CT image of the pancreatic lesion. A simple cyst of the pancreatic tail was attached to the stomach, and the wall of the cyst was enhanced (*arrows*). A distal MPD was clearly dilated, but no pancreatic tumor was detectable around the stenosis of MPD (*arrow*)

**Fig. 2:**
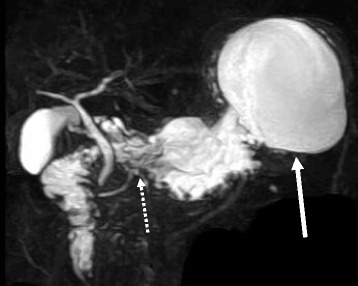
MRCP revealed a simple cyst of the pancreatic tail like CT images (*arrow*). No pancreatic tumor could be detected around the stenosis of MPD. The distal MPD was not clearly visualized (*arrow*)

**Fig. 3:**
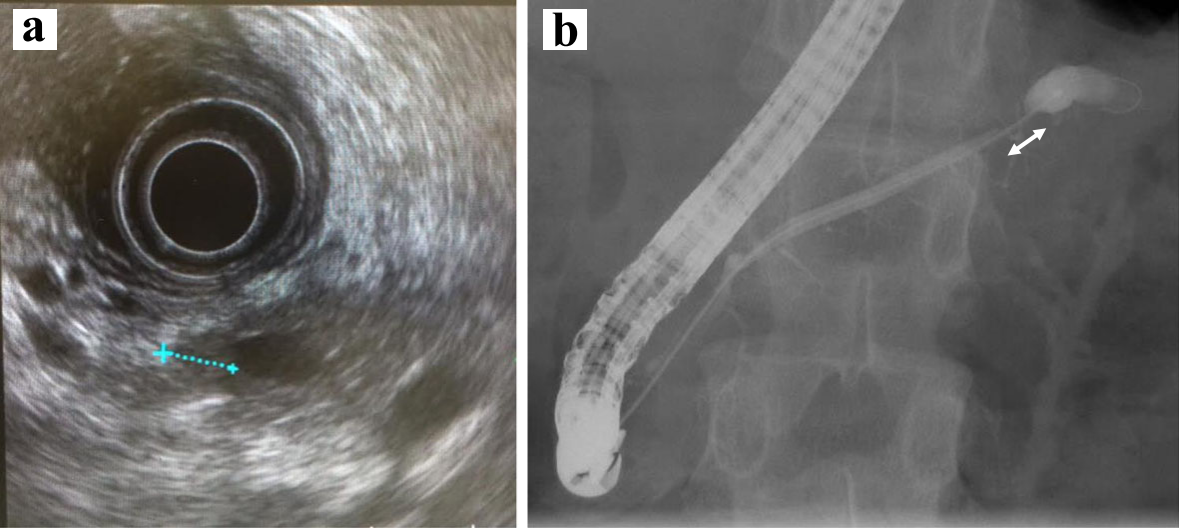
**a** EUS revealed a low density mass of 7 mm in size in the pancreatic body. **b** Endoscopic retrograde pancreatography (ERP) revealed stenosis and distal dilation of the MPD between the body and tail of the pancreas (*arrow*)

**Fig. 4:**
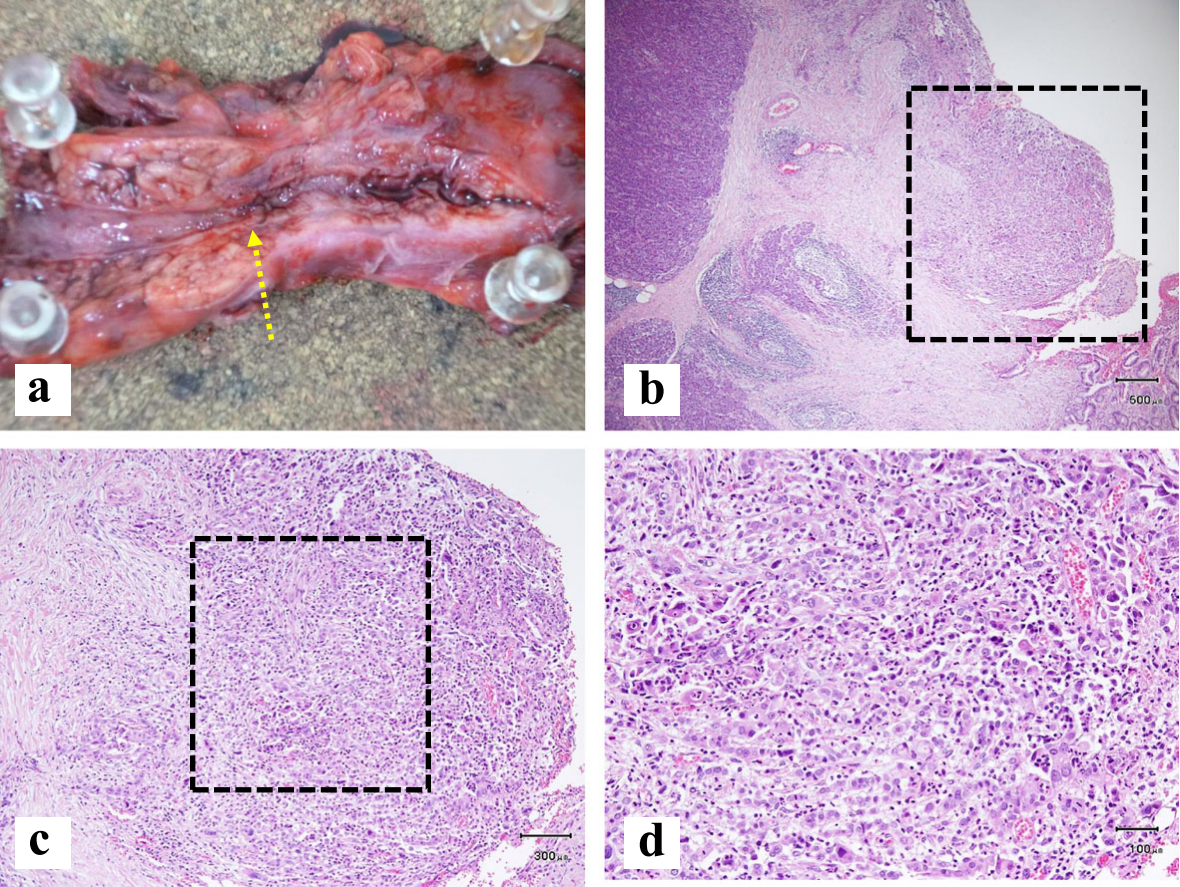
**a** On macroscopic findings, the distal MPD was obviously dilated, but the tumor could not be detected around the stenotic MPD. **b**, **c**, **d** In microscopic views, the pancreatic tumor of 7 mm in size was detected. The tumor cells were localized with proliferation of fibroblast cells in the framework. Pathological features in H&E stain

A 53-year-old male visited his primary physician for epigastric and back pain. He had neither a family history of pancreatic cancer nor a history of alcohol as well as smoking. On laboratory examinations, the levels of serum pancreatic amylase, C-reactive protein (CRP), and carcinoembryonic antigen (CEA) were up to 250 U/l, 2.1 mg/dl, and 10.2 ng/ml, respectively. Serum carbodydrate antigen 19-9 (CA19-9), DUPAN-2, and immunoglobulin G4 (IgG4) were within normal limits. Abdominal-enhanced computed tomography (CT) revealed a simple cyst of the pancreatic tail attached to the stomach (Fig. 1). The distal MPD was clearly dilated, but no pancreatic tumor was detectable around the stenosis of MPD by CT scan and magnetic resonance cholangiopancreatography (MRCP) (Fig. 2). Endoscopic retrograde pancreatography (ERP) revealed stenosis and distal dilation of the MPD located at transition between the body and tail of the pancreas. The MPD of the pancreatic head was smooth. Endoscopic ultrasound (EUS) revealed a low density mass of 7 mm in size in the pancreatic body with distal dilation of the MPD, but fine needle aspiration of the mass was not performed because of the small size and the risk of dissemination of cancer cells (Fig. 3a). After insertion of an endoscopic nasopancreatic drainage (ENPD) tube over the stenosis of MPD (Fig. 3b), the symptoms of the patient disappeared and the size of the pseudocyst was obviously reduced. The cytology of pancreatic juice was performed three times and was negative for malignancy (class II). With a suspicion of an early staged pancreatic cancer, the patient underwent distal pancreatectomy and splenectomy with lymph node dissection (D2). Intraoperatively, there was a severe adhesion encountered between the stomach and the pseudocyst. The pancreatic mass was not detected by intraoperative ultrasound. The line of pancreatic resection was above the superior mesenteric vein (SMV). Operation time and intraoperative bleeding were 275 min and 557 ml, respectively. Perioperative transfusion was not used (Fig. 4).

On histopathological evaluation, the small pancreatic adenocarcinoma of 6 mm in size was detected near the stenotic MPD. The obvious cancer cell invasion around the tumor was not detected but an invasion to the small veins (v1) and lymphatic ducts (ly1) as well as small neural infiltration (ne1) were present. Final pathological staging was moderately differentiated invasive ductal adenocarcinoma of the pancreas with no lymph node metastasis (Japan Pancreatic Society (JPS) classification 7th edition; Pbt, TS1 (6 mm), tub2, intermediate type, INF β, ly1, v1, ne1, mpd(-), pT1b, pN0, pM0, stage IA,PCM(-), DCM(-) and the Union International Control Cancer (UICC) classification of malignant tumors 6th edition; pT1, pN0, pM0, stage IA, R0). The resection margins were fine of cancer involvement. The patient developed surgical site infection and chylous ascites, and the patient was discharged on postoperative day 53. The patient will receive an adjuvant chemotherapy with TS-1 for 6 months.

## Conclusions

Previously, a small tumor (<2 cm in diameter) was a good prognotic factor in patients with pancreatic cancer for the first time in 1964 [3]. Therefore, the tumor size (TS) was categorized by the criteria of the JPS as TS1, <2 cm; TS2, 2.1–4.0 cm; TS3, 4.1–6.0; or TS4, >6.0 cm. However, the rates of T1 and stage I in the JPS classification in all patients with TS1 pancreatic cancer were only 21.6 and 14.9%, respectively, because of locally advanced tumor invasion. Moreover, the rate of lymph node metastases was 46.1% in patients with TS1 pancreatic cancer [2]. Therefore, the TS may not reflect prognosis in patients with pancreatic cancer. Other investigators suggested that the small pancreatic ductal adenocarcinoma (<1.0 cm in diameter) or carcinoma in situ was a good postoperative prognosis, thus categorized as early pancreatic cancers [4]. The rates of early pancreatic cancer, TS1 pancreatic cancer, or stage I pancreatic cancer in patients with all resectable pancreatic cancers were 0.55, 4.05, or 27%, respectively [2]. However, a significant difference of prognosis between an early pancreatic cancer and other stage I pancreatic cancers with a tumor diameter of 1–2 cm had not been reported because of the small numbers of patients.

Tumor markers such as CEA and CA19-9 are very useful tools to screen for detection and recurrences of malignant diseases. Although about 80% of patients with pancreatic cancer are positive for serum CA19-9, mostly in advanced stages, false-positive rates are very high (20–30%) in patients with beginning hepatobiliary-pancreatic diseases [5]. The estimation of CEA and CA19-9 may not be useful to diagnosis as pancreatic cancer at an early stage. However, in the current case, the preoperative serum CEA was slightly increased. After distal pancreatectomy, serum CEA decreased to normal limits. In this case, the reason of preoperative elevation with serum CEA seemed to be caused by the pancreatic pseudocyst of the tail.

The microscopic invasion of the lymph ducts and vessels in patients with pancreatic cancer is associated with significantly poor prognosis because of high T stage, absent lymph node metastasis, and perineural invasion [6]. However, a significance of microscopic invasion of the lymph ducts and vessels in patients with pancreatic cancer in stage I remains unclear.

The diagnosis of early pancreatic tumor is still difficult despite the development of CT, MRI, and endoscopic ultrasound (EUS). MRI offers several benefits for imaging of the pancreas. In particular, MRCP obtained with long echo times on T2-weighted MR images may help to demonstrate the pancreatic ductal systems and detect small pancreatic tumors. However, the differentiation between small pancreatic cancer and pancreatitis is still difficult on T1-weighted and T2-weighted MR images [7]. The preoperative indication of ERCP except for a biliary drainage has been declining because of the development of MRCP. However, ERCP seems a very useful examination to detect stenosis of the MPD in patients with small pancreatic cancer combined with inflammation as compared with MRCP [8]. In the current case, ERCP clearly detected the local stenosis of the MPD and the normal smooth MPD of the pancreatic head which suggested the negative suspicion of a pancreatitis. Therefore, diagnostic ERCP seems still valuable if a tumor is suspected despite the negative results on other images or differentiation between inflammation and cancer. EUS is reported to be superior to CT, MRI, and PET in the detection of small tumors in the pancreas and lymph node metastases as well as invasion of the major artery [9]. The typical features of pancreatic cancer was seen by EUS as a hypoechoic mass with irregular borders compared with normal pancreatic tissue. EUS-guided fine needle aspiration (EUS-FNA) is also a safe and highly accurate method for a pathological diagnosis of a tumor. However, the disadvantage of EUS as well as EUS-FNA is that the accuracy and sensitivity of these examinations depend on the technique of the operators. As a result, to detect small pancreatic tumors and for differential diagnosis of pancreatic cancer and pancreatitis, we need to consider the advantage and disadvantage of each image modality.

Nakamura T et al. [10] was reported to perform pancreaticoduodenectomy in patients with pancreatic cancer less than 1 cm in a diameter. The case was not any symptoms but incidentally serum CA19-9 elevation. To our knowledge, this is the first resectable case of such a small pancreatic cancer less than 1 cm in a diameter with complication of acute pancreatitis and formation of a pseudocyst due to obstruction of the MPD.

## References

[1] Evans DB, Abbruzzesse JL, Willett CG, De Vita VT, Hellman S, Rosenberg SA (2001). Cancer of the pancreas. Cancer-Principles and Practice of Oncology.

[2] Jung KW, Kim MH, Lee TY, Kwon S, Oh HC, Lee SS, Seo DW, Lee SK (2007). Clinicopatholpgical aspects of 542 cases of pancreatic cancer: a special emphasis on small pancreatic cancer. J Korean Med Sci.

[3] Monge JJ, Dockerty MB, Wollaeger EE, Waugh JM, Priestley JT (1964). Clinicopathologic observations on radical pancreatoduodenal resection for peripapillary carcinoma. Surg Gynecol Obstet.

[4] Ariyama J, Suyama M, Satoh K, Sai J (1998). Imaging of small pancreatic ductal adenocarcinoma. Pancreas.

[5] Sawabu N, Watanabe H, Yamaguchi Y, Ohtsubo K, Motoo Y (2004). Serum tumor markers and molecular biological diagnosis in pancreatic cancer. Pancreas.

[6] Hong SM, Goggins M, Wolfgang CL, Schulick RD, Edill BH, Cameron JL, Handra-Luca A, Herman JM, Hruban RH (2012). Vascular invasion in infiltrating ductal adenocarcinoma of the pancreas can mimic pancreatic intraepithelial neoplasia: a histopathologic study of 209 cases. Am J Surg Pathol.

[7] Jenkins JP, Braganza JM, Hickey DS, Isherwood I, Machin M (1987). Quantitative tissue characterisation in pancreatic disease using magnetic resonance imaging. Br J Radiol.

[8] Andersson R, Vagianos CE, Williamson RC (2004). Preoperative staging and evaluation of resectability in pancreatic ductal adenocarcinoma. HPB.

[9] Muller MF, Meyenberger C, Bertschinger P, Schaer R, Marineck B (1994). Pancreatic tumors: evaluation with endoscopic US, CT, and MR imaging. Radiology.

[10] Nakamura T, Masuda K, Harada S, Akioka K, Sako H (2013). Pancreatic cancer: slow progression in the early stages. Int J Surg Case Rep.

